# Unprecedented *in vivo* activity of telacebec against *Mycobacterium leprae*

**DOI:** 10.1371/journal.pntd.0013076

**Published:** 2025-05-08

**Authors:** Aurélie Chauffour, Emmanuelle Cambau, Kevin Pethe, Nicolas Veziris, Alexandra Aubry

**Affiliations:** 1 Sorbonne Université, CNRS, INSERM, Centre d’Immunologie et des Maladies Infectieuses, CIMI, Paris, France; 2 Université Paris Cité, IAME Inserm UMR 1137, Service de Mycobactériologie Spécialisée et de Référence, Laboratoire Associé du CNR des Mycobactéries et de La Résistance des Mycobactéries Aux Antituberculeux (CNR-MyRMA) APHP GHU Paris Nord, Hôpital Bichat, Paris, France; 3 Lee Kong Chian School of Medicine, Nanyang Technological University, Experimental Medicine Building, Nanyang, Singapore; 4 Singapore Centre for Environmental Life Sciences Engineering (SCELSE), Nanyang Technological University, Nanyang, Singapore; 5 National Centre for Infectious Diseases (NCID), Nanyang, Singapore,; 6 AP-HP, Sorbonne-Université, Centre National de Référence des Mycobactéries et de la Résistance des Mycobactéries aux Antituberculeux, Paris, France; Swiss Tropical and Public Health Institute: Schweizerisches Tropen- und Public Health-Institut, SWITZERLAND

## Abstract

**Background:**

New drugs targeting the electron transport chain (ETC) seem to be a promising advance in leprosy treatment. In this study, we evaluated the bactericidal activity of telacebec (TCB), a phase 2 drug candidate for tuberculosis, alongside known ETC-targeting antibiotics, bedaquiline (BDQ) and clofazimine (CFZ), as monotherapy or in combination.

**Methodology/ Principal Findings:**

We used the reference leprosy proportional bactericidal mouse footpad model. Four hundred and ten mice were inoculated in the footpads with 5x10^4^ to 5x10^0^ bacilli of *M. leprae* strain THAI53 for the untreated control group and groups treated with drug-monotherapies, and with 5x10^4^ to 5x10^1^ for groups treated with drug-combinations. Mice were randomly allocated into the following groups: 2 control groups (untreated or standard multi drug therapy (MDT), rifampin, dapsone and clofazimine with dosing equipotent to human dosing) and 7 test groups (TCB 10mg/kg, bedaquiline 25mg/kg (BDQ), clofazimine 20mg/kg (CFZ), CFZ + BDQ, TCB + BDQ, TCB + CFZ, TCB + CFZ + BDQ). Mice in the test groups received either one month treatment (MDT) or a single dose of the drugs (TCB, RIF, BDQ, CFZ). Twelve months later, mice were sacrificed to enumerate *M. leprae* bacilli in the footpad. All the footpads became negative in the MDT, TCB and combination groups except in the TCB + CFZ group where 2 mice remained positive in the 5x10^4^ inoculum.

**Conclusion:**

We demonstrated that monotherapy of TCB exhibited bactericidal activity against *M. leprae* comparable to that of MDT and that all combination therapies were as effective as MDT, except the combination TCB + CFZ, possibly due to an antagonism between these two drugs.

## Author summary

Leprosy multidrug therapy (MDT) efficacy is threatened by the rise of drug resistance. New hope arises with new antituberculous drugs targeting the electron transport chain (ETC). In this study, we evaluated the bactericidal activity of telacebec (TCB), a phase 2 drug candidate for tuberculosis, alongside known ETC-targeting antibiotics, bedaquiline (BDQ) and clofazimine (CFZ), as monotherapy or in combination. We used the leprosy proportional bactericidal mouse footpad model. Four hundred and ten mice were inoculated with *M. leprae* and randomized into control groups (untreated or MDT-treated) and treated groups (TCB, BDQ, CFZ, or combinations). Only TCB monotherapy exhibited bactericidal activity comparable to that of MDT. All combination therapies were as effective as MDT, except the combination TCB + CFZ, possibly due to an antagonism between these two drugs. The unprecedented potency of telacebec against *M. leprae* highlights its potential for further evaluation in preclinical models to guide the design of future clinical trials.

## Introduction

Leprosy, a neglected tropical disease caused by *M. leprae and M. lepromatosis*, remains a chronic disease endemic to tropical regions, disproportionally affecting the global South [1]. The physical disfigurement it causes is associated with social stigma. Despite progress in reducing the disease burden, over 200,000 new cases are detected annually across more than 120 countries, and growing resistance to current antileprosy drugs poses a significant concern [2,3]. Therefore, the development of shorter and effective regimens using novel potent bactericidal drugs is crucial.

Evaluating drug efficacy in leprosy is challenging. Although *M. leprae* was the first human bacterial pathogen identified by Hansen in 1873, it is not cultivable on artificial media [4]. In the absence of *in vitro* culture systems, animal models have become a crucial alternative for leprosy research. Despite promising molecular methods are currently being developed and evaluated to assess *M. leprae* viability, Shepard’s method (*i.e.,* the gold standard leprosy proportional bactericidal mouse footpad model) remains the reference method to assess efficacy of new drugs in a specific and quantitative manner, providing critical insights information for leprosy control efforts [5–12]. Indeed, despite the expression of *hsp18* and *esxA* transcripts by *M. leprae* correlated with bacterial growth in the Mouse Footpad (MFP) assay, it does not yet allow precise quantification of bacterial killing rates.

Historically, the discovery of antileprosy agents has been closely linked to the tuberculosis (TB) field. In recent years, following the development of the diarylquinoline bedaquiline (BDQ) and the nitroimidazoles delamanid and pretomanid, telacebec (TCB) has emerged as the third modern new drug class with proven antituberculosis activity in humans [13,14]. Among these new anti-TB drugs, bedaquiline and telacebec seem also promising against leprosy [15]. Interestingly, these two drugs, like clofazimine (CFZ) – a cornerstone of the standard MDT for leprosy - target enzymes within the electron transport chain [6,7,16–22].

BDQ inhibits the F_O_F_1_ ATP synthase by binding to the subunit c of the enzyme, leading to a decrease in bacterial ATP level [23]. Inhibition of ATP-synthesis likely has a greater impact on *M. leprae* than on tubercle bacilli as its electron transport chain and means of energy production have been extensively downsized [24,25]. In a murine model, the bactericidal activity of orally administered BDQ against *M. leprae* was comparable to that of monotherapies of either moxifloxacin or rifampin (RIF) [9]. These promising results have led to the evaluation of BDQ in a clinical trial [26,27].

TCB, also known as Q203, is a novel first-in-class antituberculosis drug that targets the mycobacterial cytochrome *bcc:aa*_*3*_ terminal oxidase complex [28]. TCB is bacteriostatic against *M. tuberculosis* due to the presence of the cytochrome bd oxidase, an alternative terminal oxidase [29]. The loss of the cytochrome bd oxidase in *Mycobacterium ulcerans* and *M. leprae* drastically sensitizes these bacteria to TCB [15,30,31].

Due to the reductive evolution of the oxidative phosphorylation pathway in *M. leprae*, we hypothesized that a drug regimen targeting different complexes could act synergistically and display a strong bactericidal activity while limiting resistance emergence [24]. Therefore, the purpose of this study was to measure the bactericidal activities of different drugs acting on the oxidative phosphorylation pathway alone and in combination in a relevant mouse model of infection.

## Methods

### Ethics statement

The experimental project was favorably evaluated by the ethics committee n°5 Charles Darwin localized at the Pitié-Salpêtrière Hospital (Paris, France). Clearance was given by the French Ministry of Higher Education and Research under the number APAFIS#30674–202103181532328 v6. Our animal facility received the authorization to carry out animal experiments (license number D75-13–08). The persons who carried out the animal experiments had followed a specific training recognized by the French Ministry of Higher Education and Research and follow the European and the French recommendations on continuous learning. The design of the experimental project followed the guidelines ARRIVE [32].

### Materials

**Fig 1:**
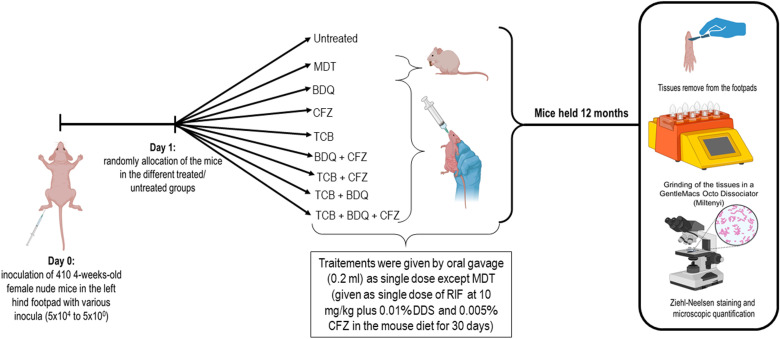
Protocol design of the study. *created with Biorender.*

Mice were infected with a *M. leprae* THAI53 strain (Fig 1). This strain was fully susceptible to the common antileprosy drugs (i.e., Rifampin (RIF), dapsone (DDS), Clofazimine (CFZ) and fluoroquinolones) [33]. The suspension used to inoculate mice was prepared from mice already infected by this isolate one year earlier. Shepard and McRae method was used to prepare the suspension [34]. Briefly, the tissue from the footpads was aseptically removed and then homogenized by using a GentleMacs Octo Dissociator (Miltenyi) under a volume of 2 ml of Hanks’ balanced salt solution. Ten µl of the prepared suspension were taken to perform Ziehl-Neelsen staining to count *M. leprae* Acid Fast Bacilli (AFB). Suspensions needed to inoculate mice were then further diluted in Hanks’ balanced salt solution.

Four-week-old nude mice (Rj:NMRI-*Foxn1*^*nu/nu*^) were purchased from Janvier Labs (Le Genest Saint Isle, France). The Rj:NMRI-*Foxn1*^*nu/nu*^ mice present an autosomal recessive mutation in the Forkead box N1 gene (Foxn1; chromosome 11) which causes a total or a partial thymic aplasia. This aplasia leads to a lack of T cells production and to the immunodeficiency of those mice. The nude mouse model aims therefore to mimic low-immunity leprosy by representing multibacillary leprosy.

DDS and CFZ were purchased at Biosynth (Slovakia), RIF at Merck (France), BDQ at LKT Labs (USA), and TCB (TELACEBEC) was provided by Kevin Pethe.

### Infection of mice with *M. leprae* and treatment

We used the proportional bactericidal method that allows measurement of the bactericidal activity of a compound [35]. Four hundred and ten 4-week-old female nude mice were infected in the left hind footpad with 0.03 ml of the *M. leprae* isolate according to Shepard’s method [36]. Mice were inoculated with four different inocula of 5x10^4^, 5x10^3^, 5x10^2^, 5x10^1^ AFB/ footpad except for 5 groups (*i.e.,* untreated control, standard MultiDrug Therapy (MDT) regimen, BDQ, CFZ and TCB groups) which were also inoculated with one 5x10^0^ additional group. Mice were randomly allocated into one untreated control group and 8 treated groups of 10 mice each: standard MDT regimen, BDQ 25 mg/kg, CFZ 20 mg/kg, TCB 10 mg/kg, CFZ + BDQ, TCB + BDQ, TCB + CFZ, TCB + CFZ + BDQ. Except for the standard regimen, treatment was given by oral gavage in a volume of 0.2 ml per mouse as a single dose the day after inoculation. To mimic the standard MDT regimen, the mice of the standard MDT regimen group were treated with a single dose of RIF 10-mg/kg plus 0.01% DDS and 0.005% CFZ in the mouse diet for 30 days. All drug dosing were selected in order to be equipotent to human dosing, *i.e.,* to generate pharmacokinetic parameters close to those in human at usual dosing or at dosing well tolerated in phase 1 or 2 trials: BDQ 25 mg/kg in mice correspond to 300 mg in human, CFZ 20 mg/kg to 100 mg and TCB 10 mg/kg to 300 mg [6,8,23,31,37–42].

### Assessment of the effectiveness of the treatment

To permit multiplication of *M. leprae* to a detectable level, mice were held 12 months in the animal facility. Mice were then euthanized and tissues from their footpad were removed aseptically and homogenized in a volume of 2 ml of Hank’s balanced salt according to Shepard’s method [36]. *M. leprae* bacilli were considered to have multiplied (*i.e.,* survived the treatment) if those footpads were found to contain ≥5.10^4^ AFB, regardless of the size of the inoculum.

### Statistical analysis

The proportion of viable *M. leprae* after treatment was determined from the infectious dose required to show multiplication in 50% of the inoculated mice. The significance of the differences between the groups was calculated by the Spearman and Kärber method [5]. A p-value <0.05 was considered statistically significant by standard evaluation. For multiple comparisons between the groups, Bonferroni’s correction was applied, *i.e.*, the difference would be significant at the 0.05 level only if the P value was adjusted to the number of groups: 0.05/n in which n was defined as the number of primary comparisons. Thus, the corrected P was 0.05/7 = 0.007 for the analysis comparing the treated groups to the untreated group and 0.05/8 = 0.0055 for the analysis comparing the MDT treated groups to the other treated groups.

## Results

### Bactericidal activity determined by the proportional bactericidal method

We first estimated the percentage of viable bacilli via calculation of the median infectious doses in mice. Twelve months after inoculation, the proportion of viable *M. leprae* bacilli in the untreated group was 1.84% of the total number of acid-fast bacilli (AFB) inoculated.

The results showed that the percentage of viable bacilli was significantly lower in all treated groups compared to the untreated one (Table 1). The percentage of bacilli killed under treatment ranged between 76.3% and ≥ 99.9% of untreated controls depending on drug or drug combination (Table 1); CFZ was the least efficacious drug.

**Table 1:** Bactericidal activity of ETC inhibitors alone or in combination against *M. leprae* THAI53 measured in nude mice by the proportional bactericidal method.

Treatment	No. of footpads showing multiplication^b^ of *M. leprae*/ No. of footpads harvested by inoculum^*^					% viable *M. leprae*^c^	% viable *M. leprae* killed by treatment^d^	P value for comparison with	
	5x10^4^	5x10^3^	5x10^2^	5x10^1^	5x10^0^	untreated control^e^	MDT^f^		
**Untreated control**	8/8	8/8	9/9	5/10	1/8	1.840	–	–	–
**MDT** ^a^	0/8	0/8	0/10	0/9	0/6	<0.0004	≥99.9	<0.001	–
**BDQ 25 mg/kg**	1/6	2/6	0/9	0/6	0/8	0.001	99.6	<0.001	0.06
**CFZ 20 mg/kg**	5/5	9/9	9/9	0/6	0/9	0.436	76.3	0.0026	0.003
**TCB 10 mg/kg**	0/9	0/5	0/9	0/6	0/7	<0.0004	≥99.9	<0.001	ns
**CFZ + BDQ**	0/2	0/8	0/9	0/9	–	<0.0004	≥99.9	<0.001	ns
**TCB + BDQ**	0/5	0/8	0/7	0/3	–	<0.0004	≥99.9	<0.001	ns
**TCB + CFZ**	2/7	0/7	0/8	0/7	–	0.001	99.9	<0.001	0.121
**TCB + CFZ + BDQ**	0/5	0/7	0/6	0/7	–	<0.0004	≥99.9	<0.001	ns

^a^ single dose of RIF at 10 mg/kg plus 0.01% DDS and 0.005% CFZ in the mouse diet for 30 days

^b^* M. leprae* bacilli were considered to have multiplied if the harvest from a footpad yielded > 5x10^4^ acid-fast bacilli

^c^ the proportion of viable *M. leprae* surviving the treatment could be calculated by estimating the “most probable number” (MPN) of viable organisms. However, the estimation of the MPN is based on the assumption that the organisms are distributed randomly in an inoculum; in the case of *M. leprae*, this assumption is probably untenable, therefore, the preferred alternative is to calculate the “median infectious dose (ID50)”, *i.e.,* the number of organisms required to infect 50% of the mice as allowed by the Spearman-Kärber method (it requires that the titration be carried out over a range of 100% to 0%). In mice, if the highest inoculum is 5x10^4^ *M. leprae* per footpad, a proportion of viable *M. leprae* as small as 0.00006 may be measured, then it is possible to calculate the proportion of viable *M. leprae* killed by the treatment by comparing the proportions of viable in treated and control mice. The significance of the differences between the groups was calculated by the Spearman and Kärber method [5]

^d^ calculated from the comparison of the proportion of viable organisms between untreated controls and the treated groups

^e^ each treated group was compared to the untreated group of mice

^f^ each treated group was compared to the group of mice treated by the MDT regimen

ns, not significant

* ten mice were initially inoculated in each group. Among all the groups, some deaths occurred due to their advanced age or technical issues (*e.g.,* gavage, cage or water supply issue).

We then compared the treated groups to the reference MDT treated group (Table 1). Among monotherapies, CFZ was the sole group significantly less effective than the standard MDT regimen. BDQ also had a lower efficacy, although the difference did not reach significance after Bonferroni correction, whereas the efficacy of TCB was comparable to that of the MDT regimen. Notably, TCB was the only single drug treatment that cleared AFB in all mouse footpads. In contrast, one mouse footpad remained positive in the BDQ group at the highest inoculum, and most of the mouse footpads were still positive for CFZ at higher inocula.

**Fig 2:**
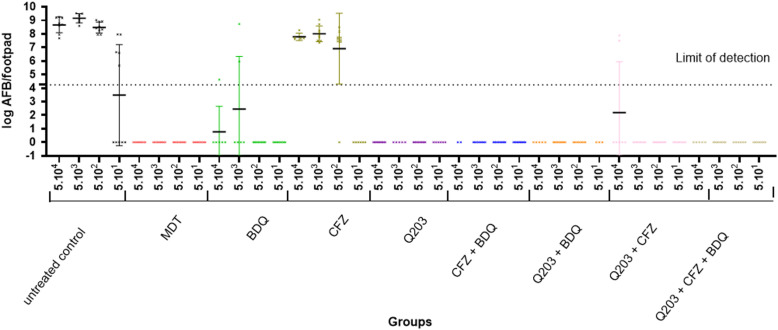
Bacterial load in nude mice infected with *M. leprae* THAI53 and treated by respiratory chain inhibitors alone or in combination (each mouse footpad is taken as a data point and the dotted line indicates the threshold of detection of *M. leprae*).

We also analyzed the AFB load per footpad for the different inocula (Fig 2 and Table 2). These results indicated a lower efficacy for CFZ and BDQ compared to the MDT regimen, while TCB matched the efficacy of the MDT regimen. Among the two-drug combinations, all showed activity comparable to the standard MDT regimen, achieving complete AFB clearance, except for the TCB + CFZ combination, where two footpads remained positive at the highest inoculum. This resulted in lower efficacy of TCB + CFZ combination compared to the MDT regimen, while the other two drug combinations were as effective as the MDT regimen.

**Table 2:** Range of AFB counts (log_10_) among positive footpads recovered from the proportional bactericidal method per inoculum.

Treatment	Range of AFB among positive footpads (mean log_10_ AFB/footpad and standard deviation)				
	5x10^4^	5x10^3^	5x10^2^	5x10^1^	5x10^0^
**Untreated control**	7.68-9.25 (8.66 ± 0.58)	8.58-9.52 (9.16 ± 0.34)	7.94-9.01 (8.47 ± 0.41)	5.66-7.96 (6.96 ± 0.99)	7.87^a^
**MDT** ^a^	<4.6^b^	<4.6^b^	<4.6^b^	<4.6^b^	<4.6^b^
**BDQ 25 mg/kg**	4.62	5.96-8.73 (7.34 ± 1.96)	<4.6^b^	<4.6^b^	<4.6^b^
**CFZ 20 mg/kg**	7.61-8.27 (7.79 ± 0.27)	7.35-9.04 (8.01 ± 0.58)	7.41-8.48 (7.77 ± 0.37)	<4.6^b^	<4.6^b^
**TCB 10 mg/kg**	<4.6^b^	<4.6^b^	<4.6^b^	<4.6^b^	<4.6^b^
**CFZ + BDQ**	<4.6^b^	<4.6^b^	<4.6^b^	<4.6^b^	
**TCB + BDQ**	<4.6^b^	<4.6^b^	<4.6^b^	<4.6^b^	
**TCB + CFZ**	7.49-7.89 (7.69 ± 0.28)	<4.6^b^	<4.6^b^	<4.6^b^	
**TCB + CFZ + BDQ**	<4.6^b^	<4.6^b^	<4.6^b^	<4.6^b^	

^a^: only one footpad positive among eight footpads, therefore the mean log_10_ AFB/footpad and standard deviation are not given.

^b^: all footpads were microscopically negative, therefore the number of AFB is below the threshold of 5x10^4^ AFB per footpad (*i.e.,* < 4.6 log_10_).

Not surprisingly, the combination of the three ETC inhibitors TCB + BDQ + CFZ achieved efficacy comparable to the MDT regimen, with no detectable AFB in any of the treated mice.

## Discussion

The current length of leprosy treatment remains a significant challenge, compounded by the growing threat of antimicrobial resistance, which jeopardizes the objective of leprosy’s elimination. This highlights the urgent need for new, more effective anti-leprosy drugs [2,3,43]. Over the past decades, few new antituberculous drugs have been discovered, with very few active against *M. leprae*. Among these, new drugs targeting the respiratory chain such as BDQ and TCB, have shown promise. Their potency is uniquely amplified in *M. leprae* by the evolutionary loss of numerous genes involved in energy production and drug efflux [24]. Indeed, *M. leprae* lacks the alternative terminal oxidase, the cytochrome bd oxidase, that maintains respiration and ensures *M. tuberculosis* survival during TCB treatment. Moreover, the absence of the MmpL5 drug efflux system, which confers low-level resistance to BDQ and CFZ in *M. tuberculosis*, *in vitro* and in patients, is another advantage for leprosy treatment [44,45]. Given that CFZ, a well-established anti-leprosy drug, also partially inhibits the ETC, we designed a study to assess the efficacy of TCB, BDQ and CFZ against *M. leprae* [6,17], both as monotherapies or in combination. This evaluation is essential for determining the potential of combining ETC inhibitors for leprosy treatment.

We demonstrated that TCB exhibits an unprecedented efficacy against *M. leprae*, surpassing BDQ at a single dose (Fig 2, Tables 1 and 2). This finding aligns with the observation in *M. ulcerans*, likely due to a comparable loss of the cytochrome bd oxidase in *M. leprae* [46]. Indeed, unlike *M. tuberculosis*, classical strains of *M. ulcerans* have a naturally occurring mutation in the *cydA* gene that renders the cytochrome bd oxidase nonfunctional[30,46,47]. Therefore, most *M. ulcerans* strains causing Buruli Ulcer (BU) are exquisitely susceptible to TCB with very low MIC values of 0.000075 to 0.00015 µg/ml [30,47]. *In vivo* studies have also shown TCB to be a very attractive candidate for treatment of BU. Scherr et al. showed that TCB alone at a daily dose of just 0.5 mg/kg was as effective as the rifampin – streptomycin (RIF – STR) combination, with 9 out of 10 mice culture-negative after 4 weeks of treatment[30]. Doses of 5, 10, and 20 mg/kg/day were also shown to be effective against *M. ulcerans* [31,48,49].

This study reaffirmed the respective good and moderate efficacy of BDQ and CFZ monotherapies against *M. leprae* in mice (Fig 2, Tables 1 and 2). We previously demonstrated that the antileprosy activity of a single oral dose of 25 mg/kg BDQ was as effective as rifapentine (10 mg/kg), moxifloxacin (150 mg/kg), and RIF (10 mg/kg), which is currently the most potent antileprosy drug, while a single oral dose of 20 mg/kg CFZ was less effective than BDQ [9–11]. It is worth noting that all these dosing evaluated are equipotent to human dosing, *i.e.,* generate pharmacokinetic parameters close to those in human (rifapentine 10 mg/kg in mice correspond to 600 mg in human, moxifloxacin 150 mg/kg to 400 mg and RIF 10 mg/kg to 600 mg) [50,51].

A key strength of the current study is the use of the standard MDT regimen as benchmark rather than RIF alone as a surrogate of the MDT treatment. This approach allowed us to demonstrate, for the first time, that although BDQ and CFZ achieved results statistically similar to MDT, neither drug could eliminate *M. leprae* in all mice, unlike TCB, which achieved complete bacillary clearance (Fig 2, Tables 1 and 2).

Another key strength of the study is the low percentage of viable bacilli that can be detected (0.004 viable *M. leprae* bacilli (Table 1)) made possible by using immunocompromised mice instead of immunocompetent ones. Indeed, it is well established that immunocompromised mice, lacking T cell immunity, support significantly higher replication of *M. leprae* and yield more viable bacilli, compared to immunocompetent mice [9]. As such, our model represents a more stringent, pessimistic replication scenario compared to the human situation.

A third strength is the use of drug dosing equipotent to human dosing for the three tested drugs, enabling direct translation of our results to humans [6,8,23,31,37,38]. Regarding the newly studied TCB, dose-proportional pharmacokinetics (PK) were observed for TCB at 2–10 mg/kg in mice, which likely corresponds to the daily doses of 100–300 mg that were reported to be well tolerated and safe in phase 1 and phase 2a trials in TB patients, provided the drug is administered with food [31,38].

The treatment of leprosy needs to be based on a combination of drugs to avoid the emergence of resistant strains. Therefore, we also evaluated the potency of combinations of ETC inhibitors. Combining BDQ and CFZ reduced the numbers of AFB positive footpads (Table 1), enabling this combination to be as bactericidal as MDT, whereas combining TCB and CFZ has led to a slight decrease in TCB activity (Table 1). This suggests an antagonism between TCB and CFZ which might be due to a competition in their mode of action or a pharmacokinetic interaction not explored in this study. Among all the studies in murine tuberculosis and Buruli Ulcer, only one has shown an antagonism between respiratory chain inhibitors [48,52]. However, this latter study failed to show any PK interaction [52]. The combination of TCB and BDQ was as effective as TCB administrated alone (Table 1) and MDT, suggesting no antagonism between these two drugs. Finally, the negative effect of CFZ observed in the TCB-CFZ combination was alleviated by the introduction of BDQ, indicating that the strong efficacy of BQD and TCB was sufficient to counteract the possible antagonism due to CFZ. Of note, the current CFZ-containing MDT regimen may lead to side effects such as hemolytic anemia, and potentially fatal hypersensitivity syndrome reactions (induced both by DDS and CFZ) and skin discoloration (induced by CFZ only). Moreover, the use of RIF is associated with drug-drug interactions, including induction of its own metabolism and metabolism of dapsone as well as medications for other conditions prevalent in Low-and Middle-Income Countries. For all these reasons, it is highly recommended to develop a safer and shorter anti-leprosy regimen [4].

Altogether, these results demonstrate that a combination of drugs targeting the ETC, especially TCB and BDQ, is highly bactericidal against leprosy, opening the way to leprosy elimination. Given these results, it is likely that TCB will form the backbone of a transformative novel MDT to achieve an unparalleled treatment shortening with potential to justify a 2-drug treatment regimen in all leprosy patients. Lack of pre-existing resistance and overall safety profile, including lack of drug-drug interactions, presents a clear advantage over MDT even post exposure prophylaxis. These findings open the path to the design of shorter TCB-based treatment deserving to be evaluated in leprosy clinical trials.
